# Paramedicine students’ perception of preparedness for clinical placement in Australia and New Zealand

**DOI:** 10.1186/s12909-015-0446-7

**Published:** 2015-10-05

**Authors:** Helen Hickson, Brett Williams, Peter O’Meara

**Affiliations:** 1 La Trobe Rural Health School, La Trobe University, PO Box 199, Bendigo, VIC 3552 Australia; 2 Department of Community Emergency Health & Paramedic Practice, Monash University, Frankston, Australia

**Keywords:** Clinical education, Clinical placements, Hospital preparation, Paramedicine, Undergraduates

## Abstract

**Background:**

Clinical placement is an essential element of paramedicine education and training as the profession completes the transition from vocational training to a pre-employment, university based model. The objective of this study was to survey pre-employment paramedicine students at Universities in Victoria, Australia and Auckland, New Zealand to measure their self-assessed preparedness for clinical placement.

**Methods:**

This was a cross-sectional study involving paper-based questionnaires employing a convenience sample of 682 undergraduate paramedicine students (years 1–4) who had completed at least one clinical placement. Student perceptions of preparedness for clinical placement were measured using an adaptation of the ‘Preparedness for Hospital Practice’ questionnaire.

**Results:**

There are significant differences in students’ perception of preparedness for clinical placement, which reflects the differences between universities in relation to structure of their paramedicine programs, the timing of clinical education and the number of hours of clinical placement.

**Discussion:**

There needs to be clinical placement agreements between the ambulance services and universities that clearly describe the standards and expected elements of a quality clinical placement.

**Conclusions:**

In order to improve the preparedness for placement for paramedicine students, a united approach is required by all stakeholders, including ambulance services, students and universities.

## Background

In Australian and New Zealand, paramedicine education has undergone significant structural changes as a university qualification becomes the pathway to the paramedicine profession [1, 2]. As vocationally based post-employment training draws to an end, there are now 17 universities in Australia and New Zealand that offer a pre-employment Bachelor degree program as a pathway to paramedicine practice [3, 4]. There is diversity in the paramedicine qualification programs that are offered in Australia and New Zealand, with undergraduate courses ranging from 3 to 4 years in duration, with options for single and double degree qualifications, post-graduate conversion programs for nurses and other health professionals, and significant variation in the focus and duration of clinical education [5]. With this diversity brings inconsistency and disparity between university providers in the preparedness of students for paramedicine clinical placement and arguable future clinical practice.

Clinical education for paramedicine students can take many different forms [6]. Internationally, there are diverse ways to approach the delivery and quality assurance of paramedic education. In the US, Canada and the UK, there are many hundreds of accredited courses, with a small number of degree programs [7]. In Australia and New Zealand there are currently no standards or requirements about the number of hours of clinical placement, where they should be located (ambulance service, hospital, simulation) nor how to measure quality. As a result, there is much variety in the number of hours of clinical education, which year of study they are linked with, whether placements are situated in the ambulance service, hospital, pre-hospital or community setting, and whether placement hours include simulated clinical experiences [5]. There is a paucity of literature about how to evaluate and measure quality in paramedicine clinical placements in any setting as clinical education literature is mostly conducted with nursing and medical students [7]. Whilst there is research in paramedicine about student perceptions of the learning environment [6–8], and how simulations can be used as an alternative for clinical education [9, 10] we were able to locate only one study [11] that directly explored paramedicine students’ preparedness for clinical placement.

The objective of this study was to survey pre-employment paramedicine students at universities in Victoria, Australia and Auckland, New Zealand to measure their self-assessed preparedness for clinical placement.

## Methods

### Design

We used a cross-sectional study involving paper-based questionnaires employing a convenience sample of undergraduate paramedicine students (years 1–4) who had completed at least one clinical placement.

### Participants

A paper-based survey was conducted with paramedicine students in Australia and New Zealand. To be included in this study, the student needed to be enrolled in a paramedicine program at one of the targeted institutions and have completed at least one clinical placement. Five institutions that offer entry-level paramedicine courses in Victoria and New Zealand were targeted: Auckland University of Technology, Australian Catholic University, Monash University, La Trobe University, and Victoria University. These locations were selected as they offered a diverse variety of clinical placement duration, content and structure. This research is part of a larger study titled: ‘Paramedicine clinical placement duration and quality variance: An international benchmarking study’ [5].

### Instrumentation

Student perceptions of preparation for clinical placement learning were measured using a survey instrument titled, ‘Preparedness for Hospital Practice’ (PHP). This survey was originally developed in Australia [12] to assess junior doctors’ perceptions of preparedness for hospital practice and has been used to assess preparedness for clinical placements amongst nursing and medical students [13–15]. There is an assumption made here that there are similarities between a student’s perceptions of their preparedness for clinical placement and perceptions about preparedness for clinical practice. We argue that as paramedicine clinical placements are conducted in the clinical practice environment, this survey instrument is an appropriate and relevant measurement tool. We were granted permission by the author to use their scale [13], and adapted the survey by changing the word ‘medical’ to ‘paramedic’ (see Table 1).

**Table 1:** Modified Preparedness for Hospital Practice

Survey items by domain: “My paramedic education prepared me to:
Subscale 1: Understanding Science
1. Understand the cellular basis of disease
2. Apply principles of basic science to clinical conditions
3. Justify drug uses on the basis of their mechanisms of action
4. Select drugs on the basis of their costs, risks and benefits
Subscale 2: Practical Skills and Patient Management
5. Record clinical data systematically
6. Carry out an efficient physical examination
7. Carry out basic clinical procedures i.e. recording blood pressure
8. Carry out basic invasive procedures i.e. intravenous catheter insertion
9. Handle medical emergencies i.e. myocardial infarction
Subscale 3: Holistic Care
10. Evaluate the impact of family factors on illness
11. Understand the interaction of social factors with disease (e.g., poverty, unemployment)
12. Appreciate the importance of a patient's cultural/ethnical and religious background

The survey assesses the perceptions of paramedicine students regarding their educational achievements and preparation for clinical placements. Questions are arranged in 8 domains that include 1) understanding science, 2) practical skills and patient management, 3) holistic care, 4) prevention, 5) interpersonal skills, 6) confidence and coping skills, 7) collaboration, and 8) self-directed learning. Overall, the survey has 8 sub-scales and 27 items, and students are asked to rate each item using a response scale that ranges from 1 to 4 (Very Inadequately = 1, Very Adequately = 4). The PHP has well established internal consistency and construct validity. Higher scores indicate higher perceptions of preparedness within the given domain. No items were reverse scored.

### Procedures

During 2013, one of the researchers visited the university campuses to administer the student surveys. At the end of a class (lecture or practical workshop) students were asked to remain in the class room and the study was explained to them. Students were able to leave if they did not wish to participate. A participant information sheet and the anonymous survey instrument were distributed to each student. All participants were aged over 18 years and consent was implied if the student completed the survey. Students completed the survey in about 10 minutes by marking their answers on the survey instrument. No follow-ups were undertaken.

### Data analysis

Data was analysed using SPSS 20 and we present frequencies demographic data. Descriptive statistics were calculated for the measures and non-parametric analyses (median scores) were used due to unequal cohort sizes (Mann–Whitney U Test; Kruskal-Wallis Test), and post-hoc testing was undertaken using a Dunn-Bonferroni correction to analyse differences in the demographics (age, year level, gender, and university). The results are statistically significant if p < 0.05.

### Ethics

This research was approved by La Trobe University Human Research Ethics Committee, reference FHEC12/182, as well as the ethics committee for each of the participating universities: Auckland University of Technology, Australian Catholic University, Monash University, Victoria University, and Ambulance Victoria and St John (New Zealand).

## Results

### Participants

Completed surveys were received from 682 paramedicine students. Most of the participating students were female and the majority were aged between 20–29 years. We surveyed students at each level of study and the full distribution of participating students can be seen in Table 2.

**Table 2:** Student demographics

		Uni 1	Uni 2	Uni 3	Uni 4	Uni 5	Total
Age	<20	20	0	36	17	7	80 (12 %)
Range	20-24	66	22	170	88	32	378 (55 %)
(years)	25-29	18	1	99	15	8	141 (21 %)
	30-34	0	1	30	3	3	37 (5 %)
	35-39	3	0	18	2	0	23 (4 %)
	40 +	2	1	16	2	2	23 (4 %)
Year of	1st year	13	0	193	0	12	218 (32 %)
Study	2nd year	50	1	128	87	35	301 (44 %)
	3rd year	46	1	48	40	5	140 (20 %)
	4th year	0	23	0	0	0	23 (4 %)
Gender	Female	71	17	202	82	30	402 (59 %)
	Male	38	8	167	45	21	279 (41 %)
	Total	109 (16 %)	25 (4 %)	369 (54 %)	127 (19 %)	52 (8 %)	682

### PHP scores by year level

Five subscales revealed statistically significant differences between year level: *Holistic Care* (p < .001), *Prevention* (p = .003), *Confidence/ Coping Skills* (p < .001), *Collaboration* (p < .001) and Self-Directed Learning (p < .001). Students in first year produced the highest median score for Holistic Care and Collaboration. These included questions such as ‘My paramedic education prepared me to: Understand the interaction of social factors with disease’ and ‘Be sensitive to the needs of other healthcare staff’.

It is important to acknowledge that year level data may be influenced by the different structure of the various paramedicine programs. For example, we are unable to tell from the data if participants in year 3 were in their final year of study, or if they were enrolled in a four-year program, a double-degree program or a conversion program (e.g. qualified paramedics who were enrolled in a Bachelor of Paramedicine program). A summary of median subscale scores and indication of significant differences by year level is presented in Table 3.

**Table 3:** Year level comparison

Report: PHP Year level									
Year		US (Factor 1)	PSPM (Factor 2)	HC (Factor 3)	P (Factor 4)	IPS (Factor 5)	CCS (Factor 6)	C (Factor 7)	SDL (Factor 8)
1st year	M*d* (IQR)	13 (11–14)	18 (16–20)	12 (9–13)	10 (9–12)	8 (7–12)	13 (12–14)	11 (9–12)	7 (7–8)
2nd year	M*d* (IQR)	14 (12–15)	18 (16–20)	10 (8–11)	10 (9–12)	8 (7–12)	12 (12–14)	10 (8–11)	7 (7–8)
3rd year	M*d* (IQR)	14 (12–15)	18 (16–20)	10 (8–11)	10 (9–12)	8 (7–12)	13 (12–14)	10 (8–11)	7 (7–8)
4th year	M*d* (IQR)	13 (11–14)	18 (16–20)	10 (8–11)	9 (8–11)	9 (8–12)	12 (11–14)	9 (7–11)	6 (6–8)
Total	M*d* (IQR)	14 (12–15)	18 (16–20)	11 (9–12)	10 (9–12)	8 (7–12)	13 (12–14)	11 (9–12)	7 (7–8)
				*p <* .0001	*p =* .003		*p <* .0001	*p <* .0001	*p <* .0001

### PHP scores by gender

One subscale revealed statistically significant differences between gender: *Prevention* (p = .001). The subscale *Prevention* included items such as: ‘My paramedic education prepared me to: Encourage patients to improve their health habits (e.g., unhealthy food, obesity, smoking…) and produced a median score M*d* = 10 (IQR = 9-12) in female students. A summary of median subscale scores and indication of significant differences by gender is presented in Table 4.

**Table 4:** Gender comparison

Report: Gender (PHP)									
Gender		US (Factor 1)	PSPM (Factor 2)	HC (Factor 3)	P (Factor 4)	IPS (Factor 5)	CCS (Factor 6)	C (Factor 7)	SDL (Factor 8)
Female	M*d* (IQR)	14 (12–15)	18 (16–20)	11 (9–12)	10 (9–12)	8 (7–12)	13 (12–14)	10 (8–11)	7 (7–8)
Male	M*d* (IQR)	14 (12–15)	18 (16–20)	12 (10–13)	9 (9–11)	9 (8–13)	13 (12–14)	11 (9–12)	7 (7–8)
Total	M*d* (IQR)	14 (12–15)	18 (16–20)	11 (9–12)	10 (9–12)	8 (7–12)	13 (12–14)	11 (9–12)	7 (7–8)
					*p =* .001				

US = Understanding Science; PSPM = Practical Skills and Patient Management; HC = Holistic Care; P = Prevention; IPS = Interpersonal Skill; CCS = Confidence Coping Skills; C = Collaboration; SDL = Self-Directed Learning.

### PHP scores by age

Two subscales revealed statistically significant differences between age: C*ollaboration* (p = 0.24) and *Self-Directed Learning* (p = 0.16). Students aged 40 years or older produced the highest median score for the subscale *Collaboration,* which included items such as: ‘My paramedic education prepared me to: Be sensitive to the needs of other healthcare staff’. Students aged 20 years or younger produced the lowest median score for the subscale *Self-Directed Learning,* which included items such as: ‘My paramedic education prepared me to: Invest time in developing my knowledge and skills’. A summary of median subscale scores and indication of significant differences by university is presented in Table 5.

**Table 5:** Age Comparison

Report: Age (PHP)									
Age Range		US (Factor 1)	PSPM (Factor 2)	HC (Factor 3)	P (Factor 4)	IPS (Factor 5)	CCS (Factor 6)	C (Factor 7)	SDL (Factor 8)
<20 years	M*d* (IQR)	14 (12–15)	18 (16–20)	12 (9–13)	10 (9–12)	9 (8–13)	13 (12–14)	11 (9–12)	6 (6–7)
20-24 years	M*d* (IQR)	14 (12–15)	18 (16–20)	10 (8–12)	10 (9–12)	8 (7–12)	13 (12–14)	10 (8–11)	7 (7–8)
25-29 years	M*d* (IQR)	14 (12–15)	18 (16–20)	12 (9–13)	10 (9–12)	8 (7–12)	13 (12–14)	11 (9–12)	7 (7–8)
30-34 years	M*d* (IQR)	14 (12–15)	18 (16–20)	12 (9–13)	10 (9–12)	7 (6–11)	13 (12–14)	11 (9–12)	7 (7–8)
35-39 years	M*d* (IQR)	12 (10–15)	18 (16–20)	12 (9–13)	10 (9–12)	9 (8–13)	12 (11–13)	11 (9–12)	7 (7–8)
40 years or older	M*d* (IQR)	13 (11–14)	18 (16–20)	12 (9–13)	10 (9–12)	12 (11–15)	12 (11–13)	12 (10–13)	7 (7–8)
Total	M*d* (IQR)	14 (12–15)	18 (16–20)	11 (9–12)	10 (9–12)	8(7–12)	13 (12–14)	11 (9–12)	7 (7–8)
								*p = .*024	*p = .*016

### PHP scores by University

All subscales revealed statistically significant differences between universities. University 3 produced the highest median scores for *Holistic Care*, *Prevention*, *Confidence/Coping Skills* and *Collaboration*. University 5 produced the highest median score for *Practical Skills and Patient Management*, and University 2 and 5 produced the highest median scores for *Interpersonal Skills*. These differences might be explained by variations in the structure, length and focus of the paramedicine program in each University. A summary of median subscale scores and indication of significant differences by university is presented in Table 6.

**Table 6:** University comparison

Report: PHP University									
University		US (Factor 1)	PSPM (Factor 2)	HC (Factor 3)	P (Factor 4)	IPS (Factor 5)	CCS (Factor 6)	C (Factor 7)	SDL (Factor 8)
Uni 1	M*d* (IQR)	13 (11–14)	18 (16–20)	10 (9–13)	9 (8–11)	7 (6–12)	11 (10–13)	9 (8–11)	6 (6–7)
Uni 2	M*d* (IQR)	13 (11–14)	18 (16–20)	10 (9–13)	9 (8–11)	9 (8–13)	12 (11–13)	9 (8–11)	7 (7–8)
Uni 3	M*d* (IQR)	14 (12–15)	18 (16–20)	12 (10–13)	10 (9–12)	8 (7–12)	13 (12–14)	11 (9–12)	7 (7–8)
Uni 4	M*d* (IQR)	14 (12–15)	18 (16–20)	10 (9–13)	9 (8–11)	8 (7–12)	12 (11–13)	9 (8–11)	7 (7–8)
Uni 5	M*d* (IQR)	14 (12–15)	19 (17–21)	9(8–12)	9 (8–11)	9 (8–13)	12 (11–13)	9 (8–11)	7 (7–8)
Total	M*d* (IQR)	14 (12–15)	18 (16–20)	11 (9–12)	10 (9–12)	8 (7–12)	13 (12–14)	11 (9–12)	7 (7–8)
		*p =* .029	*p = .*020	*p <* .0001	*p <* .0001	*p <* .0001	*p <* .0001	*p <* .0001	*p <* .0001

## Discussion

This study examined paramedicine students’ perceptions of preparedness for clinical placement in Australia and New Zealand. These results reflect the differences between Universities in relation to size of student cohort, use of simulation, timing of clinical education and how the paramedicine program connects with other parts of the university. This means that students have significantly different levels of preparedness for clinical placement. Whilst some flexibility and diversity in clinical placements is necessary, the absence of clinical placement standards or agreements has led to confusion about the expectations and essential elements of a quality clinical placement. While this paper focuses on paramedicine student clinical placements, similar issues have been identified in the medicine and nursing literature [7].

There is a general trend across year level responses that students in the earlier years of their studies report that their paramedicine education has more adequately prepared them for clinical placement. Students in year 4 report lower median scores than students in year 1 for holistic care, prevention, confidence/coping skills, collaboration and self-directed learning. This is similar to other research [11] that found that final year students feel ‘somewhat prepared’ or ‘prepared’ for the workforce and identified that more placements and variety would help them to feel more prepared. This could be due to not having clear expectations about specifically what they need to do during placement. The development of national clinical placement standards could clarify this. There are questions raised about why students in year 1 report that they are more adequately prepared for clinical placement than students in year 4. This may be due to the communication barriers between the university, the ambulance services and the student, naivety or the lack of clear expectations about what is required during the clinical placement.

Female students reported feeling more adequately prepared to encourage patients to improve their health habits that their male counterparts. Other research, [16, 17] has identified connections between gender and emotion work and suggests that it is important to understand the impact of gender in paramedic practice, including gendered attitudes towards the expression of emotion and the various elements of the paramedicine role. For example, stereotypically, women are portrayed as caring and compassionate and men are portrayed as being stoic and maintaining self-control. In paramedicine, the paramedic is required to be both caring and compassionate and also be able to operate rationally and independently. Further research is required to explore these findings and understand why female students reported feeling more adequately prepared to engage in prevention activities.

There were statistically significant differences between universities. For example, students at Uni 5 produced higher median scores in relation to understanding science, practical skills and interpersonal skills. Students at Uni 2 reported higher median scores in relation to interpersonal skills and self-directed learning, whilst students at Uni 3 reported highest median scores for holistic care and prevention. These findings are not surprising and perhaps reflect the differences between universities in relation to the structure of the paramedicine program, timing of clinical placements, and when and how case simulation is used. It may be that clinical placements that are conducted outside the ambulance service (for example, hospitals and community placements) are more intimidating than an ambulance service placement. This is an issue that has not been critically examined by the paramedicine profession in Australia or New Zealand and further research is required to explore these issues.

Age is also a factor that influences the students’ perception of preparedness for placement, with students aged 40 or older reporting the highest score for collaboration and students aged less than 20 years reporting the lowest score for self-directed learning. This connects with other writing [6, 18–21] that suggests that life experience positions the mature aged paramedicine student well for the challenges of becoming ‘work ready’. However, the reported conflict and confusion about expectations between universities and the ambulance services [5] makes it difficult to be clear about the expectations and essential elements of a quality clinical placement.

These findings are similar to the recent findings of O’Brien et al. [11] in that students report that they perceive that they are well prepared for clinical placement. Our results are in contrast to a cross-sectional study undertaken by researchers at Monash University that found a gap between theory and practice and lack of engagement for students during clinical placements [22]. However, what was missing from this research was the voice of the paramedic clinical instructor and their perceptions about the preparedness for clinical placement of paramedicine students [23, 24]. Placement learning experiences are significantly influenced by the clinical instructor [25], therefore we need to understand how they perceive the issues and use this information to influence curricula development to enhance the preparedness of paramedicine students for clinical placements. In this study, we found that age, gender and life experiences influence a student’s self-assessed perception of their preparation for placement. The next step is to examine this more carefully and to include an external assessment of both the student’s preparation for placement and the student’s achievements during placement.

Clinical placement is an important feature of paramedicine student education [4–6] and is a time when students are expected to integrate theory into practice and demonstrate their practice skills. In the absence of evidence that prescribes the optimal length, timing, location and minimum requirements of clinical placement, it appears that each university has developed a student education program that best accommodates the needs of the various stakeholders. It is not clear from this research whether there is a relationship between the length, timing and location of clinical placements and graduate attributes, but importantly provides more data for further research to be undertaken.

While it is agreed that clinical placements are an essential element of a paramedicine student’s education [8, 26–29] and there are expectations implied in the Council of Ambulance Authorities course accreditation guidelines [3], it is surprising that there are no consistent standards or agreements in place to ensure quality of placements within Australian and New Zealand ambulance services. In the US, there are many hundreds of accredited courses, with a small number of degree programs [30] and ongoing discussion about the issues related to degree level education for paramedics [31]. In the UK, standards for education and clinical placement for paramedics have recently been developed [27, 28], and discussions about the practical implications are ongoing.

Outside the discipline of paramedicine, benchmarking standards have already been developed for education and clinical placement for other health professions in Australia [7] and internationally [32]. This creates opportunities for conversation and collaboration about the broader scope of public health education for community paramedics and others moving into extended roles involving the management of patients with chronic diseases [33]. This issue is being addressed internationally, with suggestions that all health professionals should have a health education component as part of their training to challenge perceptions about the scope of practice and thinking about cross-sectorial processes and inter-professional practice in the health system [34].

For students to be prepared for placement there needs to be clinical placement standards that clearly describe the expectations and essential elements of a quality clinical placement. Paramedic clinical instructors need a framework of minimum requirements and expectations to be able to consistently measure quality in clinical placements. If the purpose of transition from on-the-job training to a pre-employment qualification is improved work readiness of graduates [11], then clinical placement agreements or agreed national standards need to be developed between the ambulance services and the universities to ensure that curricula is developed with graduate attributes that meets the needs of all stakeholders, including universities, students and ambulance services. This is an issue that has been raised repeatedly [4, 5, 7, 11, 29] but, to this point, there appears to be little advancement between key stakeholders.

### Limitations

When considering the results of this study there are potential limitations. The survey instrument ‘Preparedness for hospital practice’ [12] was originally developed for graduating medical students who were asked to retrospectively rate their medical education in regards to their preparedness for hospital practice [13]. In this study, undergraduate paramedicine students were asked about their preparedness for clinical placement, and minor changes were made to the survey to contextualise for the paramedicine profession. There are clear differences in the amount of health education that a paramedicine student and a medical student would be expected to do, and this is perhaps one area where the adapted questionnaire needs further contextualising for use with non-medical students. Although this questionnaire was not validated on the paramedicine student population, we believe our results have identified important concerns about the structure of paramedicine student clinical education. Further it is a limitation that we have not been able to reveal the identities of the participating universities and therefore make explicit connections between the findings and their course curricula.

## Conclusion

This study has explored the perceptions of preparedness for hospital clinical placements from paramedicine students in years 1–4 in Australia and New Zealand during 2013. In order to address the challenges of educating and training future paramedics in the university sector, a collaborative approach is required by all stakeholders, including ambulance services, students and universities. There needs to be clinical placement agreements or national clinical placement standards between the ambulance services and universities that clearly describe the expectations and essential elements of a quality clinical placement. These standards are already in existence in Australia and New Zealand for other health disciplines. Once these requirements are clear to all, universities can make sure that students are well prepared for their clinical placement experiences.

## Abbreviation

PHP: Preparedness for hospital practice
